# Predictive Factors of Surgical Site Infection in Prosthetic Joint Surgery: A Prospective Study on 760 Arthroplasties

**DOI:** 10.1155/2022/2150804

**Published:** 2022-09-27

**Authors:** Martina Maritati, Alessandro Trentini, Davide Chemello, Elisa Mazzoni, Carlo Cervellati, Gustavo Alberto Zanoli, Carlo Contini, Giuseppe De Rito

**Affiliations:** ^1^Department of Medical Sciences, Infectious Diseases and Dermatology Section, University of Ferrara, Via Aldo Moro, Ferrara, Italy; ^2^Orthopaedic Ward, Casa di Cura Santa Maria Maddalena, Via Gorizia, Occhiobello, Rovigo, Italy; ^3^Department of Biochemical & Specialty Surgical Sciences, University of Ferrara, Via Fossato di Mortara, Ferrara, Italy; ^4^Department of Chemical, Pharmaceutical and Agricultures Sciences, University of Ferrara, Via L. Borsari 46, 44121 Ferrara, Italy; ^5^Department of Translational Medicine and for Romagna, University of Ferrara, Via Luigi Borsari 46, Ferrara 44121, Italy

## Abstract

**Purpose:**

The success of total joint arthroplasty (TJA) has led to consistent growth in the use of arthroplasty in progressively younger patients. However, more than 10 percent of patients require revision surgery due to implant failure caused by aseptic or septic inflammation. Among the latter, surgical site infection (SSI) represents one of the worst complications of TJA, potentially resulting in the removal of the prosthesis. The aim of our study was to identify potential risk factors for SSIs in a population of patients undergoing TJA.

**Methods:**

TJA were prospectively recruited at Casa di Cura Santa Maria Maddalena from February 2019 to April 2020. Age, sex, major comorbidities, American Society of Anesthesiologists (ASA) class, length of surgery, type of surgical suture, total hospital length of stay, and clinical laboratory data were collected. The study population was then divided into two groups: Group A, normal postoperative course, and Group B, patients who developed SSI at follow-up (17-25 days).

**Results:**

25/760 (3.3%) patients developed SSIs at follow-up. Clinical and demographic parameters were not different between the two groups. Total leucocyte and neutrophil values at discharge resulted to be significatively higher in Group B compared to Group A (*p* = 0.025 and *p* = 0.016, respectively). Values of 7860/*μ*L for total leucocyte and 5185/*μ*L for neutrophil count at discharge significantly predicted the future development of SSI (AUC 0.623 and AUC 0.641, respectively; *p* < 0.05) independently from confounding factors (total leukocytes: O.R. = 3, 69 [95% C.I. 1,63-8,32]; neutrophils: O.R. = 3, 98 [95% C.I. 1,76-8,97]). Deep SSIs has been diagnosed significantly before superficial SSIs (*p* = 0,008), with a median advance of 9 days.

**Conclusion:**

Total leukocytes and neutrophils at discharge seem useful to identify a population at risk for the development of septic inflammation at the surgical site following TJA. Further studies with larger populations are needed to develop a predictive SSIs risk score that should include those variables.

## 1. Background

The number of total joint arthroplasty (TJA) procedures has steadily increased over the past decades, mainly due to demographic changes, with more aged people less willing to accept activity limitations [1]. However, more than 10 percent of patients require revision surgery due to implant failure caused by aseptic [2] or septic inflammation [3]. Among the latter, surgical site infection (SSI) represents one of the most frequent healthcare-associated infections (HAI) among orthopedic patients [4]. According to some reports, the rate of SSI secondary to TJA is expected to increase by 2% to 6.5% for hip and 6.8% for knee arthroplasty, respectively, in the next decades [5], with a consequent increase in total costs related to treatment [6].

Based on Centers for Diseases Control and Prevention (CDC) classification [7], organ spacing SSI, identified as periprosthetic joint infection (PJI), occurs in 1% to 2% of primary and in 4% of revision arthroplasties [8].

Patients affected by PJI after joint replacement procedures require prolonged antibiotic therapy, revision, or removal of the prosthesis [9] and are at higher risk of impaired functional ability.

Around two-thirds of PJI cases are caused through intraoperative inoculation of microorganisms [10] and the difficulty in their treatment depends on the ability of microorganisms to grow and persist on the implant surface and on the necrotic tissue in the form of a biofilm [11].

Depending on microbial virulence, PJI may occur early (within the first four weeks after implantation) or late (typically between three months and three years). Early infections manifest with clear signs of local and systemic of inflammation and are predominantly caused by high-virulent pathogens (e.g., *Staphylococcus aureus*, *streptococci*, and *enterococci*). Late infections present with milder symptoms such as joint pain and early loosening and are caused by low-virulent organisms (e.g., coagulase-negative *staphylococci* or *Cutibacterium* species) [12].

Conservative treatment performed with Debridement, antibiotic, and implant retention (DAIR) is allowed only if the infection is diagnosed and treated within one month after implantation of the prosthesis, or within three weeks after the onset of symptoms. To predict the outcome of PJI treated with DAIR, the KLIC-score (KLIC-score: Kidney, Liver Index surgery, Cemented prosthesis, and C-reactive protein) has been formulated as a risk stratification tool [13].

In the updated edition of the CDC guidelines on SSIs prevention [14], a whole chapter is dedicated to the prevention of PJI, highlighting the increasingly predominant role of this surgery and its complications in the immediate future. However, none of the interventions studied yielded conclusive results, pointing out the urgent need to perform new studies aiming to disclose many unsolved issues on SSIs prevention in orthopedic surgery.

The aim of our study was to identify potential risk factors for the development of SSIs in a patient population undergoing TJA, which could be used in future SSI prediction scores to guide the clinical follow-up of total joint replacement.

## 2. Materials and Methods

Patients undergoing TJA surgery (hip, knee, and shoulder) were prospectively recruited at Casa di Cura Santa Maria Maddalena (Rovigo, Italy) from February 2019 to April 2020. Strict inclusion criteria were considered for the enrolment. Severe osteoarthritis was assessed by the following criteria (at least two of the four reported below), (1) pain in the affected joint confirmed by objective examination, (2) functional limitation, (3) radiological evidence of osteoarthritis including Tonnis Classification grade II-III for hip osteoarthritis, Kellgren Classification grade III-IV for knee osteoarthritis, glenohumeral arthritis with rotator cuff tear, osteonecrosis confirmed by magnetic resonance imaging (MRI), and (4) at least 3 months of conservative therapy (drugs, physiotherapy, and hyaluronic acid) proved ineffective. In case of revision arthroplasty: diagnostic signs of aseptic implant loosening associated with pain and functional impotence. The exclusion criteria were as follows: concomitant fractures at the implant site, acquired or congenital immunodeficiency syndrome, third class obesity with a body mass index (BMI) > 40, presence of infection or phlebitis, and decompensated diabetes (glycated hemoglobin > 7, 5).

Patient demographics (age and sex), major comorbidities and medications, American Society of Anesthesiologists (ASA) class [15], length of surgery, type of surgical incision, type of surgical suture, wound dressing, and total length of hospital stay were collected from clinical and surgical records. Laboratory tests such as white blood cell count, neutrophils count, hematocrit, hemoglobin, inflammation markers such as “C-reactive protein” (CRP) and “erythrocyte sedimentation rate” (ESR), total protein, and albumin values had been recorded one week before the surgical procedure and at hospital discharge.

SSIs were defined according to the CDC diagnostic criteria [7] and classified in

*(i) Superficial*. Infection occurs within 30 days after the operative procedure and involves only skin or subcutaneous tissue of the incision.

*(ii) Deep*. Infection occurs within 1 year and involves deep soft tissues (e.g., fascial and muscle layers) of the incision.

*(iii) Organ/Space*. Infection occurs within 1 year if implant is in place and the infection appears to be related to the operative procedure and involves any part of the anatomy (e.g., organs or spaces) other than the incision opened or manipulated during the operative procedure. In arthroplasty, organ/space SSI affects the joint, the prosthesis, and the periprosthetic tissue and is identified as PJI [7].

Each enrolled patient was evaluated by an infectious disease specialist between the 17^th^ and 25^th^ day after surgery for SSIs surveillance.

The study population was then divided into two groups according to the diagnosis of SSI: Group A, patients with a normal postoperative course, and Group B, patients who developed SSI at follow-up.

For each case of SSI, the clinical symptoms, etiology, and antibiotic resistance pattern (when available), type of revision surgery (if performed), and type of antibiotic therapy administered were collected. For patients with PJI treated with DAIR, the risk of failure was calculated by the KLIC-score. Data regarding outcome and one-year follow-up were also reported.

The research was carried out in accordance with the ethical principles of the Declaration of Helsinki. The Local Ethics Committee “CESC VR-RO” approved the design of this study (Study ID n. 37370) and all participants gave their written informed consent.

### 2.1. Statistical Analysis

Normality of the distribution of continuous variables was assessed by the Kolmogorov-Smirnov test. Normally distributed variables were expressed as mean ± standard deviation and compared by *t*-test. Not normally distributed variables were expressed as median (interquartile range) and compared with Mann–Whitney *U* test. Frequencies were reported as percentage and compared by Chi-square test or Fisher's exact test in the presence of a 2 × 2 contingency table. A mixed analysis of variance (ANOVA) was used to observe whether the values measured at pre- and postoperative occasions were different between the two groups of patients. The diagnostic accuracy for total leukocytes and neutrophil's count in detecting SSI was determined by a ROC (Receiver Operator Characteristic) analysis. Youden's J Index (specificity+sensitivity-1), which maximizes the specific and sensitivity, was used to find the cut-off values. The cut-off values were used to calculate the positive predictive value (PPV), the negative predictive value (NPV), and accuracy. A multivariate logistic regression was used to determine the association between high total leukocytes (>7860 cells/*μ*L) or high total neutrophil's count (>5185 cells/*μ*L) at discharge and the presence of SSI; sex, age, and BMI were included in the analysis as possible confounding factors. The analyses were performed by SPSS (IBM) for Windows, version 25. A *p*value < 0.05 was considered significant.

## 3. Results

### 3.1. Description of the Population

During the study period, a total number of 760 TJAs were recorded: 432 were total knee arthroplasty (TKA, 56.8%), 325 were total hip arthroplasty (THA, 42.8%), and 3 were total shoulder arthroplasty (TSA, 0.4%). The analyzed surgeries were almost primary arthroplasties (*n* = 730, 96%) in contrast to revisions (*n* = 30, 4%). A total of 25 out of 760 (3.3%) SSIs were detected: 15 (60%) affected TKA and 10 (40%) THA. No SSIs were recorded following TSA and prosthetic revision surgery.

According to CDC's SSIs classification [9], 15 (60%) SSIs were classified as superficial, 6 (24%) as deep, and 4 (16%) as PJI.

Clinical and demographic characteristics of Groups A and B are summarized in Table 1.

No statistically significant differences were observed between the two groups for any of the investigated parameters. Patients in both groups were more likely to be female (Group A, 65% vs. Group B, 72%, *p* = 0.47) and severely overweight (mean BMI; Group A, 28,6 vs. Group B 30,4, *p* = 0.101). Most procedures were performed by 3 orthopaedic surgeons. Mean duration of surgery was 74 minutes for group A and 76 minutes for Group B. Mean duration of hospitalization was 13 days for both groups.

A cefazolin-based antibiotic prophylaxis was used in most cases. Patients who were allergic to cefazolin or with known risk factors for MRSA colonization received vancomycin-based antibiotic prophylaxis.

### 3.2. Laboratory Test Parameters Differ in those Patients Contracting SSI

Results from laboratory tests performed in both preoperative period and at discharge are reported in Table 2. Total leucocyte and neutrophil values at discharge were significatively higher in Group B compared to Group A (*p* = 0.025 and *p* = 0.016, respectively). Despite not reaching statistical significance (*p* = 0.087), the difference in CR *p* values between the two groups suggests that this test may play a role in defining a patient with a higher risk of developing SSI as well.

**Figure 1:**
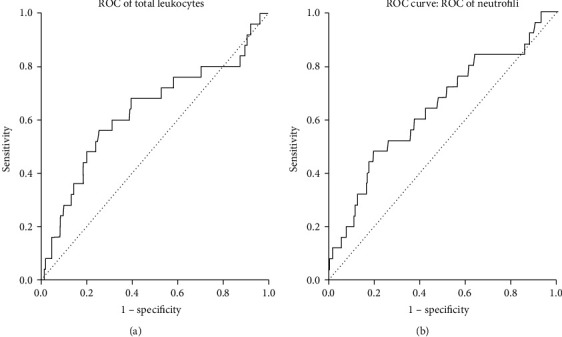
ROC curve analysis and area under the curve (AUC) calculation showing the diagnostic accuracy of total leukocyte and neutrophil's count at discharge to identify subjects at risk of developing surgical site infection (SSI). The AUC values are equal to 0.623 (*p* = 0.025) and 0.641 (*p* = 0.016), respectively.

We tested the diagnostic accuracy of total leukocyte and neutrophil's count at discharge to detect those subjects at risk to develop SSI by ROC curve analysis and area under the curve (AUC) calculation (Figure 1). AUC values were 0.623 (*p* = 0.025) and 0.641 (*p* = 0.016), respectively. By calculating the Youden's J Index, which maximizes the sensitivity and specificity, we found a cut-off value for total leukocyte of 7860 cells/*μ*L (sensitivity/specificity: 56%/75%) and for neutrophil's count of 5185 cells/*μ*L (sensitivity/specificity: 48%/80%). The two variables showed a high negative predictive value (NPV; leukocytes: 98.03%; neutrophils: 97.85%); however, they showed a poor positive predictive value (PPV; leukocytes: 6.97%; neutrophils: 7.69%).

Nonetheless, by a multivariate binary logistic regression analysis, we demonstrated that patients with values of leukocytes or neutrophils higher than the cut-off had higher odds of developing SSI, independently from confounding factors like sex, age, and BMI. In fact, patients with a value of total leucocyte at discharge higher than the cut-off had a 3.39 higher risk of developing SSI than those with lower values (O.R. = 3, 69 [95% C.I. 1,63-8,32]). Similarly, patients with neutrophils value at discharge higher than the cut-off showed a 3.98 higher risk of developing SSI than those with lower values (O.R. = 3, 98 [95% C.I. 1,76-8,97]).

**Figure 2:**
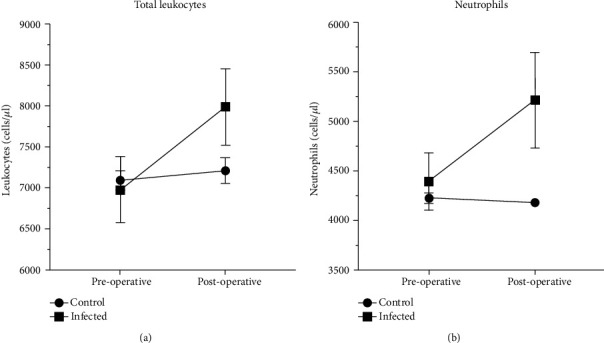
Change in total leucocytes (a) and neutrophils (b) in the two groups between preoperative and discharge measurements. As shown in Figure 2(a), patients who would develop infection (Figure 2(a), no squares) had a more pronounced increase in total leukocytes than uninfected subjects (*p* = 0.006). This interaction still exists for neutrophil's count,although it is less evident (Figure 2(b), (*p* = 0.019).

Finally, to further investigate the relation between these two parameters and the increased risk of developing SSI, we performed a mixed ANOVA analysis. By this mean, we were able to detect a SSI-related growth of values from baseline (preoperative) to discharge. As expected, the values of total leukocytes, ESR, and CRP in the whole population are increased at discharge compared to the preoperative value (*p* = 0.002, *p* = 0.001, and *p* = 0.001, respectively), whereas neutrophils almost reached statistical significance (*p* = 0.056). However, we found a significant interaction between the pre- and postoperative changes of total leukocytes and future development of infection. As displayed in Figure 2(a), patients that will develop infection (Figure 2(a), no squares) had a steeper increase in total leukocytes compared with not infected subjects (*p* = 0.006). Such interaction still exists for neutrophil's count, although it is less evident (Figure 2(b), *p* = 0.019).

### 3.3. Characteristics of SSI Classification

**Figure 3:**
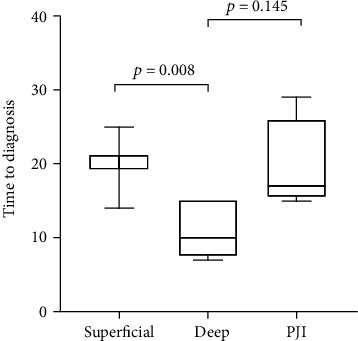
Time to diagnosis of surgical site infection (SSI) (days from the operation) in relation to the three classification categories of SSI (superficial, deep, and PJI) calculated by analysis of variance (ANOVA). Deep SSI was diagnosed significantly earlier than superficial SSI (*p* = 0.008). This difference loses statistical significance when comparing deep SSI and prosthetic joint infections (PJIs) (*p* = 0.145).

The time-to-diagnosis was compared between the three SSI classification categories (superficial, deep, and PJI) through an ANOVA analysis. As displayed in Figure 3, deep SSI was diagnosed significantly before superficial ones (*p* = 0.008), with a median advance of 9 days. This difference has loose statistical significance when comparing deep SSI and PJIs (*p* = 0.145).

According to the clinical presentation of SSI following arthroplasty, dehiscence of the surgical wound occurred in 10 patients (40%), 8 patients (32%) developed fever, and 8 cases (32%) required surgical revision. Three out of four cases (75%) of PJI were treated with DAIR; a KLIC score < 4 was registered in all cases with an expected failure rate between 4.5% and 19.4%.

A two-stage revision was carried out only in one case (0.25%).

Twelve cases of SSI (48%) needed a new hospital admission and parenteral antibiotic therapy; a microbiological diagnosis was disclosed only in 9 cases out of 25 (36%), from synovial fluid and tissue samples (*n* = 8) and bloodstream (*n* = 1), respectively.

In 5 cases (56%) the etiology of SSI has been attributed to Gram-positive organisms, in 3 (33%) cases to Gram-negative ones. A polymicrobial infection was detected only in one case (11%). The most frequently isolated bacterium was *S. epidermidis* (40%), methicillin, and fluoroquinolone resistant. Isolated pathogens and resistance patterns are shown in Table 3.

All patients completed a full course of antibiotic therapy (2 weeks for superficial/deep SSIs and 10-12 weeks for PJI). None of the patients with superficial or deep SSIs relapsed during a one-year follow-up. Two out of 4 PJIs (50%) relapsed after a conservative DAIR approach (patients relapsed at 40 days and 3 months from the end of the therapy, respectively); relapsed PJI infections were caused by MSSA and *P. mirabilis.*

## 4. Discussion

Septic inflammation following TJA represents the most frequent and expensive infectious complication in Europe and USA, as they often require prolonged hospitalization and sometimes new surgery, significantly impacting on mortality and morbidity [16].

In the present study, we enrolled a relatively large cohort of patients (*n* = 760) undergoing TJA (hip, knee, and shoulder) at Casa di Cura Santa Maria Maddalena, a third level regional reference center for orthopedic surgery.

The comparison between our study population and the Italian epidemiological data available [17] shows a lower percentage of deep SSIs and PJI compared to the national infection rates. In fact, in the Italian case series, 54% of SSIs diagnosed in orthopedic surgery are classified as deep or PJI, while in our population only 40% of SSIs match this classification.

It is worth noting that none of the revision surgeries carried out at our institution developed a SSI, although literature data points out that the incidence of this complication is significantly higher following a revision compared to a primary implant [18]. The reason for this difference, however, could be found in the small number of revisions performed (4%), compared to primary arthroplasty (96%).

Both sex and mean age of the study population are demographically homogeneous with the results reported in the “National Surveillance System of SSIs” [17]; the mean BMI of the enrolled population is 29.10 kg/m^2^, explaining how obesity plays a key role in joint arthrosis.

In the comparison of the two study groups, none of the clinical variables considered reached statistical significance. According to literature data, some of the considered variables (eg., BMI, diabetes, steroid and anticoagulant therapy, number of surgeons, duration of surgery, and staples) have been associated with an increased risk of developing SSI, an association not found in our study.

On the contrary, the analysis of the main laboratory tests brought some significant results: the total leukocytes and neutrophils counted at the time of hospital discharge were significantly higher in group B (*p* = 0, 025; *p* = 0,016, respectively). To our knowledge, the role of total leukocytes and neutrophils at discharge has never been highlighted in literature data as an independent risk factor or the development of SSI following joint replacement.

Cut-off values for total leukocytes > 7860/*μ*L and neutrophils > 5185/*μ*L may be able to discriminate the two groups. Although the diagnostic value of these tests appears weak to recommend them as diagnostic tests for SSI, their high negative predictive values (total leukocytes: 98.03%, OR: 3.69; neutrophils: 97.85%, OR: 3.98) suggest their potential role during the postoperative screening, aimed at highlighting patients with a higher risk of developing SSI, alone or as a part of a cluster of variables or a predictive score.

The limit of these two tests lies in the very low positive predictive value (total leukocytes: 6.97%; neutrophils: 7.69%), which suggests the need to integrate further the diagnostic exams in the selected population to discriminate septic inflammation from false positive cases. However, given the crucial one-month deadline to perform a successful DAIR, we believe that rapidly excluding patients at lower risk and concentrating efforts on others could be a useful (and potentially cost-effective) solution in the clinical pathway of TJA follow-ups.

By comparing the timing of SSI diagnosis to the CDC classification [7], we found an earlier SSI diagnosis for deep infections compared to the superficial ones (*p* = 0.008) and PJI (*p* = 0.145) (Figure 3). Even though in the latter case statistically significance is not reached, a relationship between the time of SSI diagnosis and the tissue depth of the infection itself has not been described yet in literature reports, except for PJI that rises later [7].

The outpatient examination performed by an infectious disease specialist for SSI surveillance was carried out between the 17^th^ and 25^th^ day postsurgery. Data from the enrolled population show that all diagnosed superficial SSI and PJI fall within this observation period. Deep SSI, on the other hand, anticipated the screening period, since many cases were diagnosed before the hospital discharge.

According to the clinical presentation, hyperpyrexia was recorded mainly in patients with deep or periprosthetic SSI.

Among SSIs with microbiological diagnosis (*n* = 9), 4/9 cases were supported by a Gram-negative bacterium. This element suggests that contamination of the surgical site may have occurred at a time other than the surgical act, presumably due to wrong management of the dressing after discharge. Risk factors for the development of SSIs from Gram-negative bacteria are diabetes mellitus and the presence of urinary catheter [19]; none of the patients who developed SSI caused by Gram-negative bacteria were diabetic, whereas data regarding the placement of a urinary catheter were not collected in this study.

All patients treated for SSIs have completed the planned therapeutic course, without any systemic or local complications. To date, no recurrence has occurred in superficial and deep SSI. The only cases of relapse involved periprosthetic infection treated with DAIR (2/4, 50%) caused by MSSA and *P. mirabilis*, respectively.

In the literature, DAIR treatment for PJI shows success rates varying between 32 and 100% [20]. DAIR represents an attractive surgical modality for the treatment of PJI, although protocols differ in several retrospective series and randomized controlled or prospective trials on this topic are lacking. It is well known that the increase in the chances of success of this procedure depends on a careful selection of patients associated to the radicality of the surgical toilette.

In the present study, the KLIC score used to stratify the risk of relapse in patients undergoing DAIR has shown poor accuracy for outcome prediction even in patients defined at “low risk” (KLIC score < 4); according to our opinion, the main limit of the score is represented by the lack of a microbiologic parameter. Relapsed periprosthetic infections, in fact, were sustained by virulent pathogens (MSSA and *P.mirabilis*, respectively).

Main limitation of our study is represented by the poor PPV of total leukocytes and neutrophils at discharge that may discourage its application as possible clinical tests to discriminate patients at risk to develop future SSI. This may be partially due to the low sample size of the group of patients presenting with SSI, which unbalanced the study. However, we consider it as a good starting point for studies with larger populations to replicate our findings.

## 5. Conclusions

Two clinical variables, total leukocytes and neutrophils at discharge, seem useful in identifying a population at risk for developing septic inflammation of surgical site after TJA. Further studies in larger populations are needed to develop a predictive SSIs risk score that should include those variables. In addition, our case series, although limited, seems to discourage the use of KLIC score in its present form as a risk stratification tool in DAIR. Additional investigations are needed to validate a successful prognostic score for DAIR procedures that considers the inclusion of bacterial-related variables, such us type of isolated strains and antimicrobial resistance profiles, when available.

## Figures and Tables

**Table 1:** Demographics and clinical variables for controls and cases.

	Group A (*n* = 735)	Group B (*n* = 25)
Sex (female; *n*, %)	478 (65)	18 (72)
Age (years ± SD)	71.2 ± 9.5	68.5 ± 13.7
BMI (kg/m^2^)	28.6 ± 5.1	30.4 ± 6.7
Diabetes (%)	13.3	9.0
Number of operators		
2	29.0	24.0
3	56.0	56.0
4	14.7	20.0
5	0.1	0.0
Prosthesis type (%)		
Hip	42.8	40.0
Knee	56.7	60.0
Shoulder	0.5	0.0
Cemented (yes, %)	57.8	60.0
Antibiotic (%)		
Cefazolin	94.0	92.0
Vancomycin	6.0	8.0
ASA score (mean ± SD)	2.6 ± 0.5	2.6 ± 0.5
Duration of surgery (minutes; mean ± SD)	74 ± 19	76 ± 17
Steroid drugs (%)	4.4	4.0
Anticoagulant therapy (%)	31.2	31.8
Hospitalization (days, mean ± SD)	13.0 ± 2.9	13.0 ± 2.8
Type of suture (%)		
Clip	14.7	20.0
Absorbable stitches	85.3	80.0

SD: standard deviation.

**Table 2:** Laboratory parameters measured before the surgical procedure and at discharge.

	Control (*n* = 735)		Infected (*n* = 25)	
	Preoperative	Discharge	Preoperative	Discharge
Preoperative serum albumin	4.6 [4.4-4.8]	—	4.5 [4.2-4.7]	—
Preoperative CRP	17.0 [7.0-37.0]	86.0 [40.0-157.0]	21.0 [5.5-59.5]	123.0 [69.5-176.0]
Pre-operative ESR	16.0 [9.0-28.0]	45.0 [30.0-62.0]	12.0 [6.0-25.0]	43.0 [27.0-63.5]
Total leukocytes (cells/*μ*L)	6690 [5720-8020]	6720 [5770-7870]	7030 [5560-8545]	7970 [6235-9680]^a^
Neutrophils count (cells/*μ*L)	4010 [3200-4930]	3880 [3160-4920]	4320 [3305-5100]	4900 [3650-5885]^b^

Values are reported as median [interquartile range]; SD: standard deviation; CRP: C-reactive protein; ESR: erythrocyte sedimentation rate. ^a^*p* = 0.025 vs. control; ^b^*p* = 0.016 vs. control.

**Table 3:** Isolated pathogens and their resistance pattern.

Pathogen	Number of isolates *n* (%)	Tested antibiotic	Resistance (%)
*S. epidermidis*	4 (40%)	Methicillin	100%
		Fluoroquinolones	100%
*P. mirabilis*	2 (20%)	Fluoroquinolones	0%
		Cotrimoxazole	50%
*S. aureus*	1 (10%)	Methicillin	0%
		Fluoroquinolones	0%
		Cotrimoxazole	0%
*S. agalactiae*	1 (10%)	Fluoroquinolones	0%
*P. aeruginosa*	1 (10%)	Fluoroquinolones	0%
*E. cloacae*	1 (10%)	Fluoroquinolones	0%

## Data Availability

The dataset on which this paper is based is too large to be retained or publicly archived with available resources. Documentation and methods used to support this study are available from Dr. Martina Maritati at Orthopaedic Ward, Casa di Cura Santa Maria Maddalena, Via Gorizia, Occhiobello, Rovigo, Italy.

## Abbreviations

TJA: Total joint arthroplasty
SSI: Surgical site infection
HAI: Healthcare-associated infections
PJI: Periprosthetic joint infection
BMI: Body mass index
CDC: Centers for diseases control and prevention
DAIR: Debridment, antibiotic, implant retention
MRI: Magnetic resonance imaging
ASA: American society of anesthesiologists
CRP: C-reactive protein
ESR: Erythrocyte sedimentation rate
ANOVA: Analysis of variance
TKA: Total knee arthroplasty
THA: Total hip arthroplasty
TSA: Total shoulder arthroplasty.
